# Ca^2+^ influx through muscle-type nicotinic acetylcholine receptors in zebrafish contributes to contractions and development of slow muscle cells in early development

**DOI:** 10.1098/rsob.250226

**Published:** 2025-11-26

**Authors:** Buntaro Zempo, Fumihito Ono, Koichi Nakajo

**Affiliations:** ^1^ Division of Integrative Physiology, Department of Physiology, Jichi Medical University, Shimotsuke, Tochigi, Japan; ^2^ Division of Life Sciences, Department of Physiology, Osaka Medical and Pharmaceutical University, Takatsuki, Osaka, Japan

**Keywords:** zebrafish, nicotinic acetylcholine receptor, skeletal muscle, neuromuscular junction, muscle contraction

## Abstract

Although the difference between the characteristics of fast and slow muscles has been extensively studied, it is still not fully understood. Here, we propose that nicotinic acetylcholine receptors (AChRs) expressed in slow muscles of zebrafish have high Ca^2+^ permeability compared to that of AChRs of fast muscles. To analyse the significance of the Ca^2+^ influx through AChRs in slow muscles, we generated a transgenic (Tg) zebrafish line that expresses Ca^2+^-impermeable AChRs in its slow muscles. The locomotor activities of the Tg zebrafish were markedly decreased at 1–3 days post-fertilization (dpf) compared to those of zebrafish expressing Ca^2+^-permeable AChRs in their slow muscles. Ca^2+^ imaging suggested that Ca^2+^ influx via AChRs is crucial for the Ca^2+^ response during muscle contraction in 2 dpf larvae, as slow muscle cells of the Tg line lacked a sustained Ca^2+^ response. Furthermore, we found that slow muscles of the Tg line became thinner compared to those expressing Ca^2+^-permeable AChRs. These short Ca^2+^ responses and thinner slow muscles may have induced locomotion impairment in the Tg line. These results suggested the physiological roles of the Ca^2+^ influx through AChRs in slow muscles and provided new insights into the characterization of fast and slow muscles.

**Figure:**
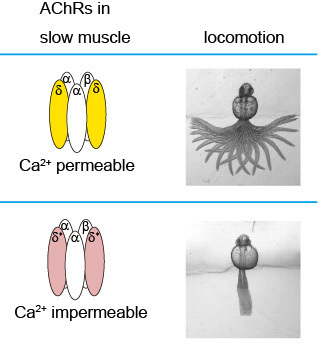

## 1. Introduction

Muscle contraction mechanisms have long been studied and are well understood [1–3]. Neuromuscular junctions (NMJs) play crucial roles in the process of muscle contraction. At the NMJs, motor neurons release acetylcholine (ACh) and stimulate nicotinic acetylcholine receptors (AChRs). AChRs are ligand-gated cation-permeable ion channels. The binding of ACh induces cation influx through AChRs, depolarizes the membrane potential, activates the voltage-gated Na^+^ channels (Nav) in the sarcolemma, and generates an action potential. Depolarization of the sarcolemma opens the L-type Ca^2+^ channels that are in physical contact with ryanodine receptors (RyRs). As the L-type Ca^2+^ channels open, RyRs also open and release Ca^2+^ from the sarcoplasmic reticulum (SR), which induces muscle contractions. In this process, AChRs are essential molecular components that play a key role in the initial step, receiving signals from motor neurons.

Muscle-type AChRs are pentamers composed of two α1s, β1, δ and ε (or γ in early development) subunits [4]. AChRs in NMJs tend to be highly permeable to Na^+^, whereas they show relatively low permeability for Ca^2+^ [5,6]. However, recent studies have revealed a different type of AChR composed of only α1, β1 and δ subunits in zebrafish [7–9]. It was also found in those studies that slow muscles and fast muscles specifically express αβδ-type and αβδγ/ε-type AChRs, respectively. This finding begs the next question: why do slow muscles express AChRs that are different from those of fast muscles?

To address this question, we focused on the difference between channel properties of slow and fast muscle-type AChRs. Although αβδ-type AChRs have been reported only in zebrafish, the larval tunicate *Ciona* has been shown to possess AChRs composed of only three types of subunits: α1, B2/4 and BGDE3 [10]. The slow muscle-type AChRs of zebrafish show characteristics similar to those of the AChRs of *Ciona*: both AChRs exhibit inward rectification [7,10]. This property is unique and not found in muscle-type AChRs described to date, including fast muscle-type AChRs of zebrafish.

On the other hand, neuronal AChRs (for example, receptors composed entirely of α7 subunits, or heteromeric receptors formed by α4 and β2 subunits) show inward rectification and high Ca^2+^ permeability [11,12], which are bestowed by negatively charged amino acid residues of the ‘intermediate ring’ (part of the ion permeation pathway of AChRs). Nishino *et al.* showed that muscle-type AChRs of *Ciona* display inward rectification and high Ca^2+^ permeability. Moreover, the intermediate rings of AChRs of *Ciona* are composed of glutamate (E), a negatively charged amino acid [10]. The authors further showed that *Ciona* AChRs lose the rectification and Ca^2+^ permeability by mutating E of the intermediate ring to glutamine (Q) (uncharged amino acid).

In zebrafish, fast muscle-type AChRs are composed of α1, β1, δ, ε or γ subunits, among which ε and γ subunits possess Q at the intermediate ring. In contrast, intermediate rings in slow muscle-type AChRs are composed of only E, because γ and ε subunits are absent.

Based on these facts, we hypothesized that slow muscle-type AChRs in zebrafish are highly permeable to Ca^2+^ and that the Ca^2+^ influx through AChRs may contribute to the functions of slow muscles. The results of the present study suggested that the slow muscle-type AChRs of zebrafish actually have high Ca^2+^ permeability. We further analysed the physiological significance of the Ca^2+^ permeability.

## 2. Material and methods

### 2.1. Maintenance of fish lines

Zebrafish were kept in water at 28°C with 14 h of light (08.00–22.00) and 10 h of darkness (22.00–08.00). Animal experiments using zebrafish were approved by the Animal Experiment Committee of Jichi Medical University (approval no. 23013-01, 23013-02).

### 2.2. Electrophysiology

cDNAs for the muscle AChR subunits α1, β1, δ and ε were previously cloned from zebrafish [7]. The cDNA of each subunit was subcloned into the pTNT vector (Promega, WI, USA) for *in vitro* transcription. The purified plasmid was linearized at the BamHI site and *in vitro* transcribed with T7 RNA polymerase (mMESSAGE mMACHINE T7 Transcription kit; Thermo Fisher Scientific, MA, USA). Oocytes were isolated and defolliculated by treatment with 2 mg ml^−1^ collagenase (Sigma-Aldrich, C0130, MO, USA) for 4–6 h in MBSH solution (88 mM NaCl, 1 mM KCl, 2.4 mM NaHCO_3_, 10 mM HEPES, 0.3 mM Ca(NO_3_)_2_, 0.41 mM CaCl_2_, 0.82 mM MgSO_4_, pH 7.6). For the expression of the α1β1δε receptor, the cRNA solution of each subunit was mixed to final concentrations of 0.2, 0.1, 0.1 and 0.1 ng nl^−1^, and 50 nl of the solution was injected per oocyte. For the expression of αβδ, cRNAs were mixed at 0.2, 0.1 and 0.2 ng nl^−1^. Injected oocytes were incubated at 17°C for 2–3 days in MBSH solution supplemented with 0.1% penicillin–streptomycin (Sigma-Aldrich). Electrophysiology was performed with a two-electrode voltage clamp using OC-725C (Warner Instruments, CT, USA). Generation of voltage-clamp protocols and data acquisition were performed using a Digidata 1550 interface (Molecular Devices, CA, USA) and Clampex 10.7 software (Molecular Devices). All experiments were performed at room temperature. A glass electrode with a resistance of 0.2–0.5 MΩ was prepared from a borosilicate glass capillary (GC150TF-10, MA, USA) using a micropipette puller (P-1000, Sutter Instrument, CA, USA). The glass electrode was filled with 3 M KCl. Low Ca^2+^ solution or high Ca^2+^ solution was used as the bath solution. Low Ca^2+^ solution consisted of (in mM) 5 HEPES, 10 NaCl, 2 KCl, 1.8 CaCl_2_, 1 MgCl_2_ and 86 NMDG, pH 7.5, and high Ca^2+^ solution consisted of 5 HEPES, 10 NaCl, 2 KCl, 10 CaCl_2_ and 1 MgCl_2_, 69.6 NMDG, pH 7.5. An ionic current was induced by puff applying 20 µM acetylcholine diluted in bath solution to the oocytes. The current–voltage (I–V) relationship was examined by applying a ramp pulse.

### 2.3. Generation of a transgenic line

We designed a gene construct that expresses a mutated δ subunit and enhanced green fluorescent protein (EGFP) under the regulation of a slow muscle-specific promoter, *psmyhc1* [13]*.* The coding sequence of the mutated δ subunit, P2A and EGFP were cloned into the Tol2 plasmid [14]. The plasmid was injected along with transposase mRNA into one-cell stage embryos. The established transgenic (Tg) line was crossed with the *sofa potato* (sop) mutant zebrafish line, and an *smyhc1*: mutated δ-P2A-EGFP; sop^-/-^ line *(pure slow Ca^2+^-imperm)* was generated.

### 2.4. Immunohistochemistry

After generating the Tg line, *pure slow Ca^2+^-imperm*, we confirmed the specificity of EGFP expression in slow muscle cells by labelling slow muscle fibres and EGFP. First, 3 days post-fertilization (dpf) larvae of the Tg line were deeply anaesthetized with 0.03% MS-222 (Sigma-Aldrich). Then, the trunk regions of the fish were fixed with 4% paraformaldehyde (PFA) in phosphate-buffered saline (PBS). The fixed samples were frontally cryosectioned at 20 μm using a cryostat (CM 3050S; Leica Microsystems, Wetzlar, Germany) and mounted onto a micro slide glass (CREST; Matsunami, Osaka, Japan). Sections were incubated overnight with a mixture of anti-EGFP antibody raised in rabbits (Thermo Fisher Scientific, MA, USA) that was diluted 1 : 500 with phosphate-buffered saline containing 0.3% Tween 20 (PBST) and F59 antibody raised in mice (DSHB, Iowa, USA) that was diluted 1 : 500 with PBST for 2 h and then rinsed twice with PBST and incubated for 2 h with a mixture of CoraLite488-conjugated goat anti-rabbit IgG (Proteintech, Illinois, USA) diluted 1 : 500 with PBST, anti-mouse IgG H&L (Alexa Fluor 555; Abcam, Cambridge, UK) diluted 1 : 500 with PBST and DAPI (Nacalai, Kyoto, Japan) diluted 1 : 2000 with PBST. The sections were then washed with PBST and coverslipped with CC/Mount (Diagnostic BioSystems, Pleasanton, CA, USA). Fluorescence was observed with a confocal microscope (Dragonfly; OXFORD INSTRUMENTS, Oxford, UK).

For the labelling of slow muscle fibres in whole-mount samples, 2 or 5 dpf larval zebrafish were fixed in 4% PFA at 4℃ for 2 h and then washed several times in PBST. The samples were then incubated in PBS containing 1 mg ml^−1^ collagenase (Sigma-Aldrich) for 60–90 min at room temperature and permeabilized with acetone for 10 min at −20℃. After washing with PBST, the samples were incubated overnight at room temperature with F59 antibody diluted 1 : 20 in PBST. Embryos were rinsed in PBST and incubated with anti-mouse IgG H&L (Alexa Fluor 555; Abcam) diluted 1 : 500 in PBST for 2 h at room temperature.

Fluorescence was observed and acquired using a camera (Digital Sight 10; Nikon, Tokyo, Japan) on an MVX10 microscope (OLYMPUS, Tokyo, Japan) and a confocal microscope (Dragonfly; OXFORD INSTRUMENTS). The data were analysed with ImageJ.

### 2.5. Locomotor analysis

High-speed image capturing of larval zebrafish was performed with a Photron camera (INFINICAM; Photron, Tokyo, Japan) at 1000 frames s^−1^. Captured images were saved as JPEG files and processed with ImageJ and software for motion analysis (Mova-tr/2 and Wriggle Tracker; Library, Tokyo, Japan). For each of the larvae (2–5 dpf), the head was touched gently with an eyelash [15] to induce escape behaviour. For 1 dpf embryos, the head and yolk were embedded in an agarose gel, and spontaneous activities were recorded. The tail bend angle, swimming speed, tail beat speed and maximum tail bend angle were calculated using Mova-tr/2 and Wriggle Tracker. Kinetics of tail bend angles were drawn by plotting degrees of tail bend angles against time. The frame at 0 ms was set immediately preceding the detection of the first motion.

### 2.6. Ca^2+^ imaging

We designed a gene construct that expresses a red fluorescent Ca^2+^ sensor protein, jRGECO1a [16] (obtained from Addgene #61563), under the regulation of a skeletal muscle-specific promoter, *pactc1b* [17]*.* The promoter activity of *pactc1* is stronger than that of a slow muscle-specific promoter *(psmyhc1).* Thus, to make observation easier, we chose the *pactc1b* promoter instead of the slow muscle-specific promoter. The coding sequence of jRGECO1a and the sequence of *pactc1b* were cloned into the Tol2 plasmid. We injected the constructed plasmid into one-cell stage embryos from AChR γ subunit^−/−^ ε subunit^+/−^ pairs and *pure slow Ca^2+^-imperm (sop^+/-^)* pairs. The genotype of the injected embryos was determined by sequence analyses after Ca^2+^ imaging.

jRGECO1a-expressing fish at 2 or 5 dpf were embedded in the lateral position in 2% low-melting agarose in a 35 mm dish. The heads were touched gently with glass pipettes to induce swimming. Ca^2+^ responses were recorded with a Zyla 4.2 sCMOS camera (OXFORD INSTRUMENTS) on an MVX10 microscope (OLYMPUS) at 200 Hz for 3 s. The data were analysed with ImageJ. Δ*F*/*F* was calculated for each frame using the formula (*F* − *F*_0_)/*F*_0_, where *F* represents the fluorescence in that frame and *F*_0_ is the resting intensity before the Ca^2+^ rise. The experiments were performed for slow muscle cells of the *pure slow Ca^2+^-imperm* and those of the γ/ε subunits double KO line (*pure slow Ca^2+^-perm*).

For *in vitro* Ca^2+^ imaging, fish at 2 or 5 dpf were deeply anaesthetized with 0.03% MS-222 (Sigma-Aldrich). After dissecting the trunk region into several pieces with surgical knives, tissue samples were incubated in 10 mg ml^−1^ collagenase solution in a bath solution (112 mM NaCl, 2 mM CaCl_2_, 3 mM Glu, 2 mM KCl, 1 mM MgCl_2_, 5 mM HEPES, pH 7.4) at room temperature for 30 min. MgCl_2_ was used to replace CaCl_2_ in a Ca^2+^-free solution. After washing twice with the bath solution, the samples were incubated in Rhod-4 solution (Abcam, Cat: ab112157) following the manufacturer’s instructions at room temperature for 30 min. Then, the samples were washed with the bath solution and placed in a recording chamber filled with the bath solution. Ca^2+^ responses were induced by puff application of 30 µM ACh using a glass pipette. Ca^2+^ responses were recorded with a Zyla 4.2 sCMOS camera (OXFORD INSTRUMENTS) on an MVX10 microscope (OLYMPUS) at 200 Hz for 14 s. The data were analysed with ImageJ. Δ*F*/*F* was calculated for each frame using the formula (*F* − *F*_0_)/*F*_0_, where *F* represents the fluorescence in that frame and *F*_0_ is the resting intensity before the Ca^2+^ rise. The peak amplitude of Δ*F*/*F*_0_ and decay time to 50% of the peak amplitude were calculated. In the present study, we adopted 50% as the reference value because the Ca^2+^ responses in muscle cells did not exhibit clear exponential decay. The experiments were performed for slow muscle cells of the *pure slow Ca^2+^-imperm* and those of the *pure slow Ca^2+^-perm*.

### 2.7. Statistical analysis

Unpaired Student’s *t*‐tests (two-tailed) were performed for statistical analysis. Averages and SEM are displayed in bar graphs.

## 3. Results

### 3.1. Acetylcholine receptors of slow muscles show high Ca^2+^ permeability

**Figure 1.**
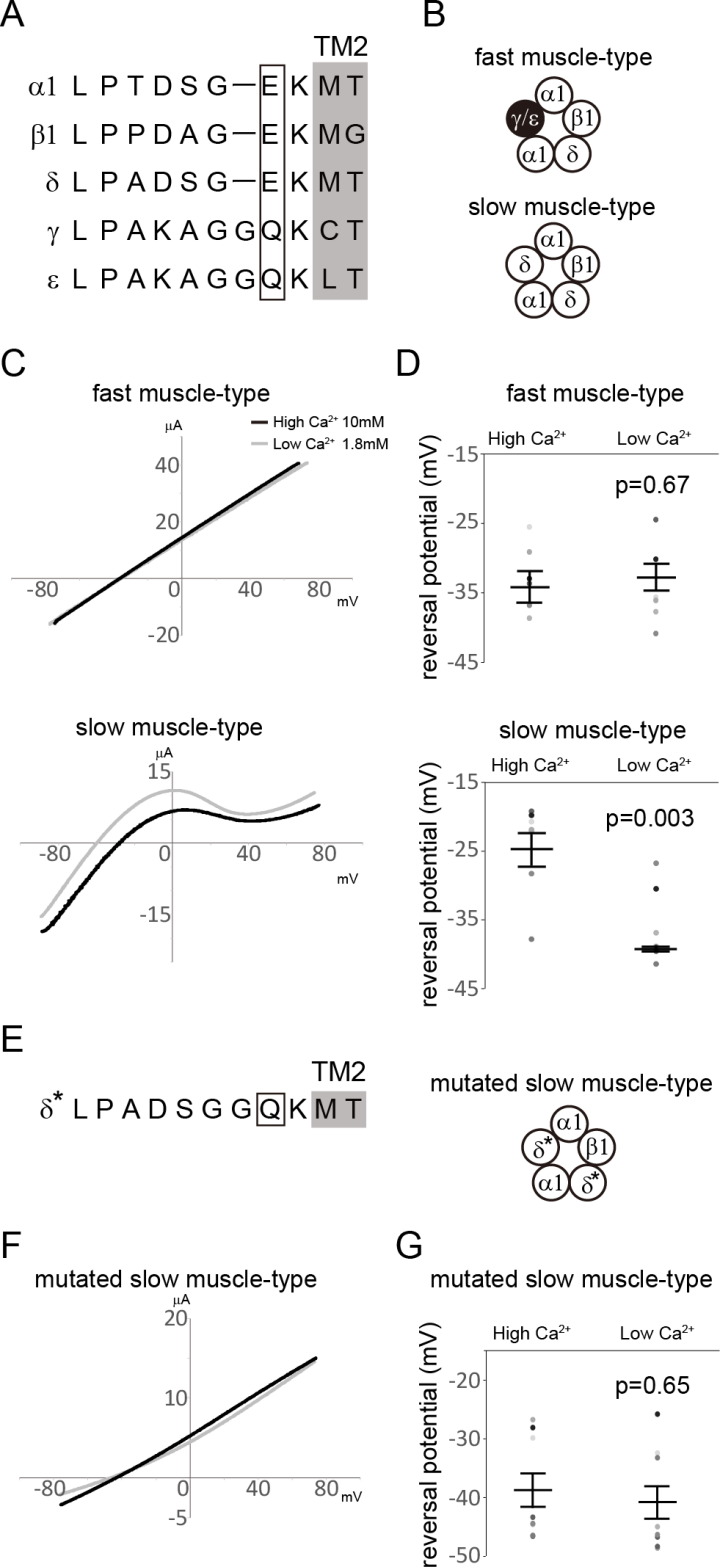
(A) Sequence alignment around the intermediate ring. Sequences of α1, β1, δ, γ and ε are shown. Amino acids that compose the intermediate ring are boxed. (B) Schematic illustrations of subunit compositions of muscle-type nicotinic acetylcholine receptors (AChRs) of zebrafish. Slow muscle cells express γ/ε-less AchRs. (C) Current–voltage (I*–*V) relationship for acetylcholine (ACh)-induced currents through fast muscle-type or slow muscle-type AChRs with external Ca^2+^ concentration of 10 mM (black lines) or 1.8 mM (grey lines). Slow muscle-type AChRs-mediated current showed inward rectification. (D) Changes in reversal potential in response to external Ca^2+^ level. In slow muscle-type AChRs, reversal potential was significantly shifted with changes in the external Ca^2+^ level. On the other hand, reversal potential did not change in fast muscle-type AchRs. (E) Sequence alignment around the intermediate ring of the mutated δ subunit (asterisk). An amino acid that composes an intermediate ring is boxed. Glu (E) of the intermediate ring is mutated to Gln (Q). A schematic illustration of the subunit composition of mutated slow muscle-type AChRs is shown on the right. (F) I–V relationship for ACh-induced currents through mutated slow muscle-type AChRs in 10 mM Ca^2+^ solution (black line) or 1.8 mM Ca^2+^ solution (grey line). (G) Changes in reversal potential in response to external Ca^2+^ level. In mutated slow muscle-type AChRs, reversal potential was not significantly shifted by changes in the external Ca^2+^ level.

First, we compared the sequence alignment of subunits that compose zebrafish muscle-type AChRs (figure 1A). The γ and ε subunits possess a glutamine (Q) residue in the intermediate ring. Fast muscle-type AChRs comprising α1, β1, δ, ε or γ subunits (figure 1B), therefore, contain one Q at the intermediate ring. On the other hand, slow muscle-type AChRs lack γ/ε-subunits (figure 1B), and their intermediate rings are composed only of glutamate (E), as in the case of AChRs of *Ciona*, which show high Ca^2+^ permeability [10].

Based on these observations, we analysed the Ca^2+^ permeability of fast muscle-type and slow muscle-type AChRs of zebrafish by electrophysiology. We recorded ACh-induced ionic currents mediated by AChRs expressed in *Xenopus laevis* oocytes by a two-electrode voltage clamp and measured reversal potential with different Ca^2+^ concentrations (1.8 and 10 mM Ca^2+^). The I–V relationships of the AChRs revealed that slow muscle-type AChRs showed inward rectification, whereas fast muscle-type AChRs showed no rectification (figure 1C). While the reversal potential of fast muscle-type AChRs was unaffected by external Ca^2+^ level (−34.1 ± 24 mV in 1.8 mM Ca^2+^, −32.7 ± 2.0 mV in 10 mM Ca^2+^; *p* = 0.67), the reversal potential significantly changed in slow muscle-type AChRs (figure 1D) (−39.3 ± 0.48 mV in 1.8 mM Ca^2+^, −25.2 ± 2.5 mV in 10 mM Ca^2+^; *p* = 0.003). To confirm the importance of amino acid residues in the intermediate ring for Ca^2+^ permeability, we mutated E of the intermediate ring in the δ subunit to Q (figure 1E). We recorded ACh-induced ionic currents mediated by AChRs that are composed of α1, β1 and mutated δ subunits. Mutated slow muscle-type AChRs showed a linear I–V relationship (figure 1F), and its reversal potential was not affected by the external Ca^2+^ concentrations (figure 1G) (−40.8 ± 3.0 mV in 1.8 mM Ca^2+^, −38.8 ± 2.9 mV in 10 mM Ca^2+^; *p* = 0.65).

### 3.2. Generation of a transgenic zebrafish line that expresses acetylcholine receptors with low Ca^2+^ permeability in its slow muscle

**Figure 2.**
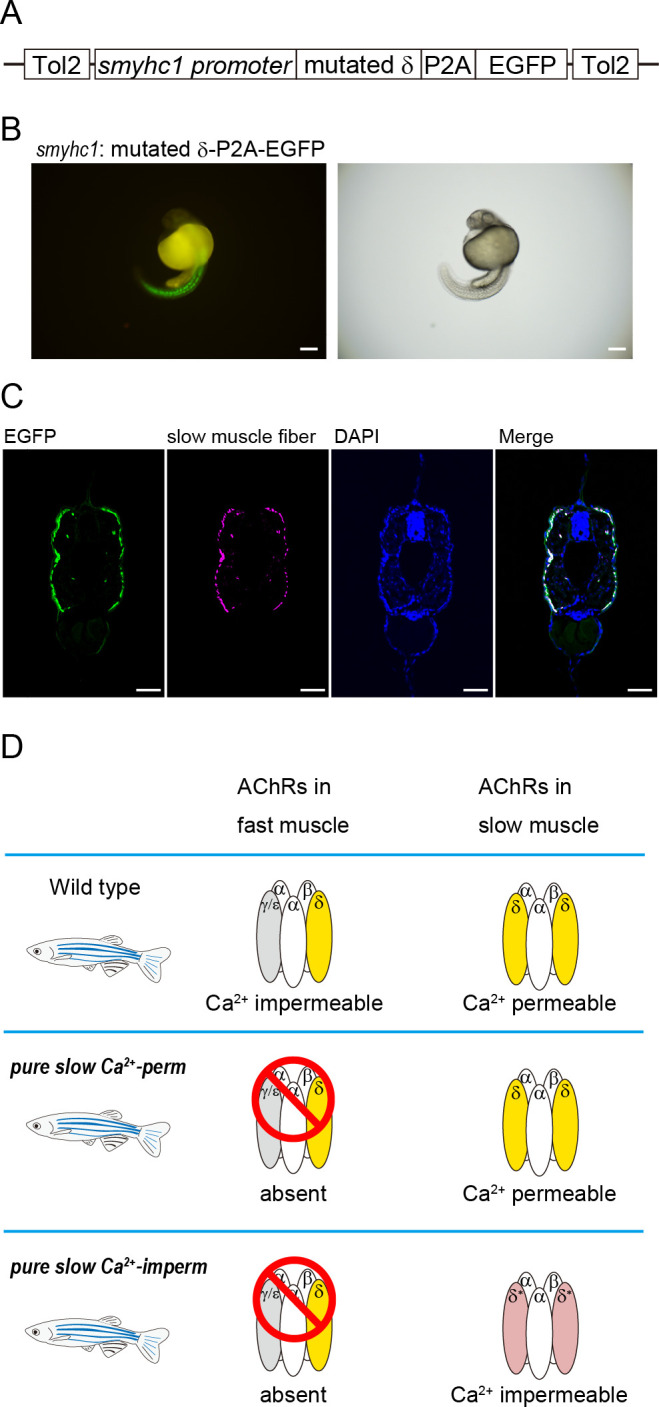
(A) DNA construct used for making a transgenic line that expresses the mutated δ subunit and EGFP under the control of a slow muscle-specific promoter sequence. (B) Tg[*smyhc1*:mutated δ-P2A-EGFP] embryo at 1 dpf. The right panel shows a bright-field image. Scale bars: 200 µm. (C) Photographs showing double immunohistochemistry for EGFP (green) and slow muscle fibres (magenta, F59 antibody) in a frontal section of the trunk region of a 3 dpf larva, which indicate that EGFP and mutated δ subunits are specifically expressed in slow muscle cells. Scale bars: 50 µm. (D) (Top) Wild-type zebrafish with AChR subunits in fast and slow muscles illustrated. (Middle) The *pure slow Ca^2+^-perm* line expresses native AChRs only in slow muscles because they lack γ/ε subunits necessary in fast muscles. (Bottom) The *pure slow Ca^2+^-imperm* fish express mutated (Ca^2+^-impermeable; shown in pink) AChRs in their slow muscles. To remove the effects of endogenous δ subunits in this Tg line, we expressed the transgene in the *sofa potato* (*sop*) background, which carries a mutation in the δ subunit gene leading to failure of assembly of AChRs in all muscle cells. Therefore, only subunits carrying the transgenic subunits are expressed in their slow muscles.

To clarify the physiological roles of Ca^2+^ influx through slow muscle-type AChRs, we generated a Tg zebrafish line that expresses slow muscle-type AChRs with low Ca^2+^ permeability in its slow muscle cells. The mutated δ subunit (figure 1E), P2A and EGFP sequences were fused to the promoter region of the slow myosin heavy chain 1 (*smyhc1*) gene (figure 2A). In the Tg line, EGFP signals were observed in trunk regions (figure 2B).

We first examined the specificity of EGFP signals by double labelling. EGFP and slow muscle fibres were visualized by anti-EGFP antibody and anti-skeletal muscle myosin antibody (F59), respectively (figure 2C). F59 antibody is widely used for detecting slow muscle cells in zebrafish. The obtained image confirmed the specificity of EGFP expression in slow muscle fibres, suggesting that slow muscle cells specifically express mutated δ subunits.

Moreover, to remove the effects of endogenous δ subunits and fast muscle-type AChRs, we crossed the Tg line and the *sofa potato* (*sop*) mutant line. In its δ subunit gene, *sop* harbours a point mutation [18], and previous studies showed that the *sop* mutant completely lacks both slow muscle-type and fast muscle-type AChRs [19]. By crossing the newly established Tg and *sop*, we generated a zebrafish line that expresses mutated slow muscle-type AChRs with low Ca^2+^ permeability in its slow muscles, while fast muscle cells lack AChRs (figure 2D). In this article, we call this line harbouring an E-Q mutated δ subunit in the *sop* background *pure slow Ca^2+^-imperm*.

To clarify the physiological importance of Ca^2+^ permeability of slow muscle-type AChRs, we compared locomotor activities of *pure slow Ca^2+^-imperm* with those of another mutant, γ/ε subunits double KO (γεDKO) [8]. The latter line expresses AChRs only in slow muscles. Of note, the γεDKO line expresses intact slow muscle-type AChRs that allow Ca^2+^ permeation. In this article, we call γεDKO *pure slow Ca^2+^-perm.* Comparison of *pure slow Ca^2+^-perm* and *pure slow Ca^2+^-imperm* was therefore expected to reveal the physiological significance of Ca^2+^ influx through slow muscle-type AChRs (figure 2D).

Both *pure slow Ca^2+^-perm* and *pure slow Ca^2+^-imperm* lines begin to lose body mass around 7–8 dpf and die by approximately 14 days.

### 3.3. Ca^2+^ influx through acetylcholine receptors plays a vital role in the locomotor activities of slow muscles at early developmental stages

**Figure 3.**
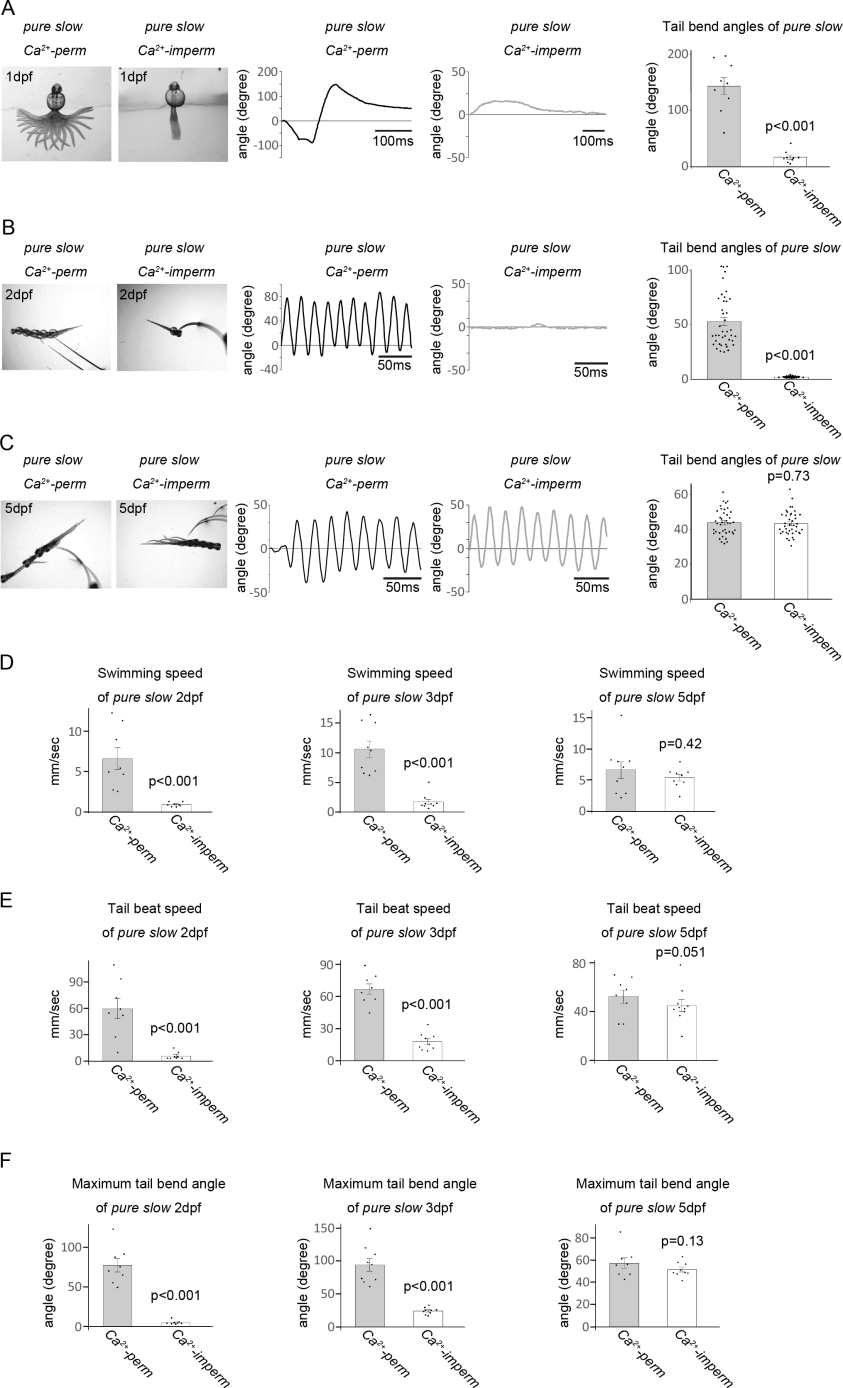
(A) Spontaneous locomotor activities of 1 dpf γεDKO (*pure slow Ca^2+^-perm*) and Tg (*pure slow Ca^2+^-imperm*) lines. Images of representative larvae on the left show superimposed frames of the spontaneous activity. The head and yolk of the embryos were partially restrained in an agarose gel, leaving the trunk and tail free to move. Kinematics for representative traces of larvae are shown in the middle panels. Tail bend angles are shown in degrees, with 0 indicating a straight body and positive and negative values indicating tail bends in opposite directions. In the right panel, maximum turn angles were calculated for each group of fish (*n* = 9 fish). (B,C) Touch-evoked startle responses of 2 or 5 dpf *pure slow Ca^2+^-perm* and *pure slow Ca^2+^-imperm* larvae. A startled response was elicited by touching the head with an eyelash. Images of representative larvae on the left show superimposed frames of the startle response. Kinematics for representative traces of larvae are shown in the middle panels. In the right panel, average turn angles were calculated for each group of fish (*n* = 40: five turns from eight fish). In 1–2 dpf larvae, the tail bend angles of *pure slow Ca^2+^-imperm* were significantly smaller than those in *pure slow Ca^2+^-perm*. However, at 5 dpf, tail bend angles of *pure slow Ca^2+^-imperm* improved to the same level as that of *Ca^2+^-perm*. (D–F) Swimming speed, tail beat speed and maximum turn angles were calculated for each group of fish (2–5 dpf). In the 2–3 dpf *pure slow Ca^2+^-imperm* line, the swimming speed, tail beat speed and maximum tail bend angle were notably reduced. However, at 5 dpf, these values improved to be comparable to those of *pure slow Ca^2+^-perm*.

Using a high-speed camera, we analysed locomotor activities of the *pure slow Ca^2+^-perm* (γεDKO) line and the *pure slow Ca^2+^-imperm* line at 1, 2, 3 and 5 dpf. At the 1 dpf stage (24–26 h post fertilization), spontaneous tail movement (coiling) was recorded (figure 3A), and we analysed tail bend angles, measured as the angle of the trunk deviation from the longitudinal axis of the head. Kinematics for representative traces showed that tail bend angles of *pure slow Ca^2+^-imperm* were smaller than those of *pure slow Ca^2+^-perm* (figure 3A). Maximum tail bend angles in *pure slow Ca^2+^-imperm* were significantly smaller than those in *pure slow Ca^2+^-perm* (142.5 ± 15.0° in *Ca^2+^-perm*, 16.8 ± 10.8° in *Ca^2+^-imperm*; *p* < 0.001). We also compared the number of coilings over 30 s between wild-type and *pure slow Ca^2+^-imperm* at 1 dpf. Since no significant difference was observed (3.5 ± 0.7 in wild-type, 2.7 ± 0.6 in *pure slow Ca^2+^-imperm*; *p* = 0.4), motor neurons in the *pure slow Ca^2+^-imperm* may stimulate the slow muscles at a frequency comparable to that of the wild-type.

At the 2 dpf stage, we induced an escape response by tactile stimuli and recorded the locomotor activity with a high-speed camera, analysing tail bend angles (figure 3B). The *pure slow Ca^2+^-perm* fish showed weak but significant swimming behaviour despite the lack of fast muscle activity (figure 3B). On the other hand, as shown in electronic supplementary material video S1, the *pure slow Ca^2+^-imperm* line wagged its tail only slightly. Kinematics for representative traces suggested that the amplitude of the tail bend angle is much smaller in *pure slow Ca^2+^-imperm* than in *pure slow Ca^2+^-perm* (see also electronic supplementary material videos S1 and S2). Calculated average tail bend angles of *pure slow Ca^2+^-imperm* were significantly smaller than those of *Ca^2+^-perm* (53.1 ± 3.9° in *Ca^2+^-perm*, 2.0 ± 0.1° in *Ca^2+^-imperm*; *p* < 0.001).

We also analysed escape behaviour at the 5 dpf stage (figure 3C). At this stage, unexpectedly, tail beat kinematics of *pure slow Ca^2+^-imperm* larvae appeared to be almost identical to those of *pure slow Ca^2+^-perm*, displaying no significant difference in average tail bend angles (43.6 ± 1.2° in *Ca^2+^-perm*, 43.1 ± 1.1° in *Ca^2+^-imperm*; *p* = 0.73, see also electronic supplementary material videos S3 and S4).

We also calculated swimming speed, tail beat speed and maximum tail beat angle in 2–5 dpf larvae (figure 3D-F). As a result, tail beat speed and amplitude were decreased in 1–3 dpf *pure slow Ca^2+^-imperm*, resulting in slower swimming. However, at the 5 dpf stage, there was no difference in locomotor functions between *pure slow Ca^2+^-perm* and *Ca^2+^-imperm*. These results suggest that the Ca^2+^ permeability of AChRs is important for the contraction process of slow muscle cells at early developmental stages.

### 3.4. Ca^2+^ imaging suggests that Ca^2+^ influx through acetylcholine receptors contributes to a sustained Ca^2+^ response of slow muscle cells in early developmental stages

**Figure 4.**
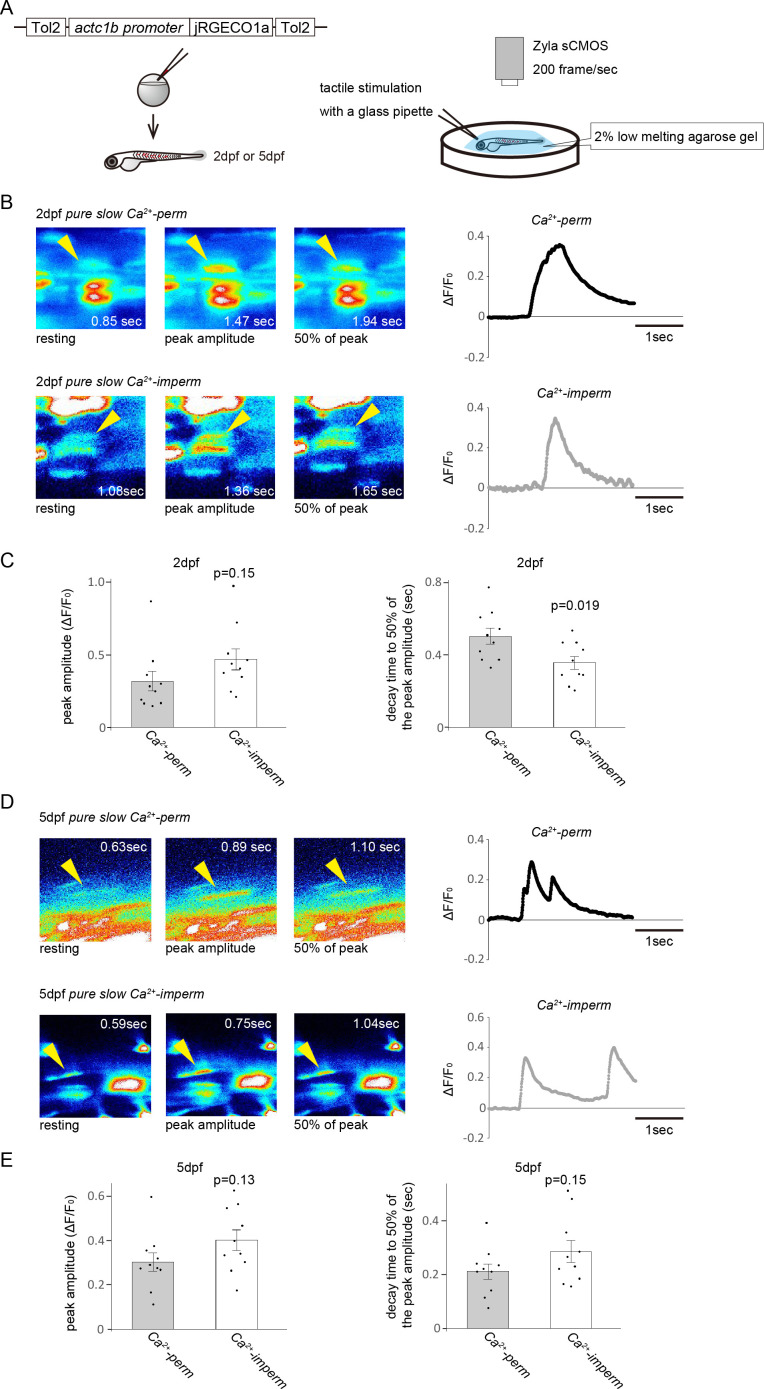
(A) Ca^2+^ response elicited by tactile stimulation in a single slow muscle cell was analysed with the Ca^2+^ sensor protein jRGECO1a. The transgenic construct expresses jRGECO1a under the control of a skeletal muscle-specific promoter sequence. The construct was injected into an embryo at the one-cell stage. Ca^2+^ response to tactile stimulation was recorded using an sCMOS camera at 200 frames s^−1^. (B) The pseudo-colour panels represent changes in fluorescence intensity of slow muscle cells of 2 dpf *pure slow Ca^2+^-perm* and *Ca^2+^-imperm*. Arrowheads indicate one of the target slow muscle cells expressing jRGECO1a. The other cells include fast muscle cells, which are silent in those zebrafish lines. Consecutive panels show the peak fluorescence intensity before, during and after the peak. Representative traces showing the increase of Δ*F*/*F*_0_ in slow muscle cells (cells indicated by arrowheads in the left panels) from *pure slow Ca^2+^-perm* (black trace) and *Ca^2+^-imperm* (grey trace). (C) The peak amplitude of Δ*F*/*F*_0_ and decay time to 50% of the peak amplitude were calculated. The decay time was significantly shorter in *pure slow Ca^2+^-imperm* than in *Ca^2+^-perm*. There was no significant difference in the peak amplitude. (D) The pseudo-colour panels represent changes in fluorescence intensity of slow muscle cells of 5 dpf *pure slow Ca^2+^-perm* and *Ca^2+^-imperm*. Arrowheads indicate slow muscle cells expressing jRGECO1a. Consecutive panels show the peak fluorescence intensity at rest, at the peak amplitude and 50% of the peak. Representative traces show the increase of Δ*F*/*F*_0_ in slow muscle cells (cells indicated by arrowheads in the left panels) from *pure slow Ca^2+^-perm* (black trace) and *Ca^2+^-imperm* (grey trace). (E) The peak amplitude of Δ*F*/*F*_0_ and decay time to 50% of the peak amplitude were calculated. There was no significant difference between the two lines in either peak amplitude or decay time.

To analyse the physiological roles of Ca^2+^ influx through AChRs, we examined the Ca^2+^ response to tactile stimulation *in vivo* with a fluorescent Ca^2+^ sensor protein (jRGECO1a) (figure 4A). We injected a construct containing a skeletal muscle-specific promoter sequence and jRGECO1a into one cell-stage embryos from *pure slow Ca^2+^-perm* pairs or *pure slow Ca^2+^-imperm* pairs and carried out Ca^2+^ imaging. At the 2 dpf stage, fluorescence traces showed that the Ca^2+^ response of slow muscle cells in *pure slow Ca^2+^-imperm* decreased faster than that of slow muscle cells of *pure slow Ca^2+^-perm* (figure 4B). We calculated the peak amplitude of the Ca^2+^ response (Δ*F*/*F*_0_) and the decay time to 50% of the peak amplitude (figure 4C). There was no significant difference in peak amplitude between *Ca^2+^-perm* and *imperm* (0.318 ± 0.068 in *Ca^2+^-perm*, 0.47 ± 0.072 in *Ca^2+^-imperm*; *p* = 0.15)*.* However, decay time was shorter in *pure slow Ca^2+^-imperm* (0.50 ± 0.044 sec in *Ca^2+^-perm*, 0.36 ± 0.036 s in *Ca^2+^-imperm*; *p* < 0.05, *p* = 0.019). We also analysed 5 dpf larvae (figure 4D-E). In the 5 dpf stage, the peak amplitude of *Ca^2+^-imperm* was not significantly different from that of *Ca^2+^-perm* (figure 4E; 0.30 ± 0.13 in *Ca^2+^-perm*, 0.40 ± 0.046 in *Ca^2+^-imperm*; *p* = 0.13)*.* In addition, there was no difference in decay time between *Ca^2+^-perm* and *imperm* (figure 4E; 0.21 ± 0.028 s in *Ca^2+^-perm*, 0.29 ± 0.040 s in *Ca^2+^-imperm*; *p* = 0.15).

**Figure 5.**
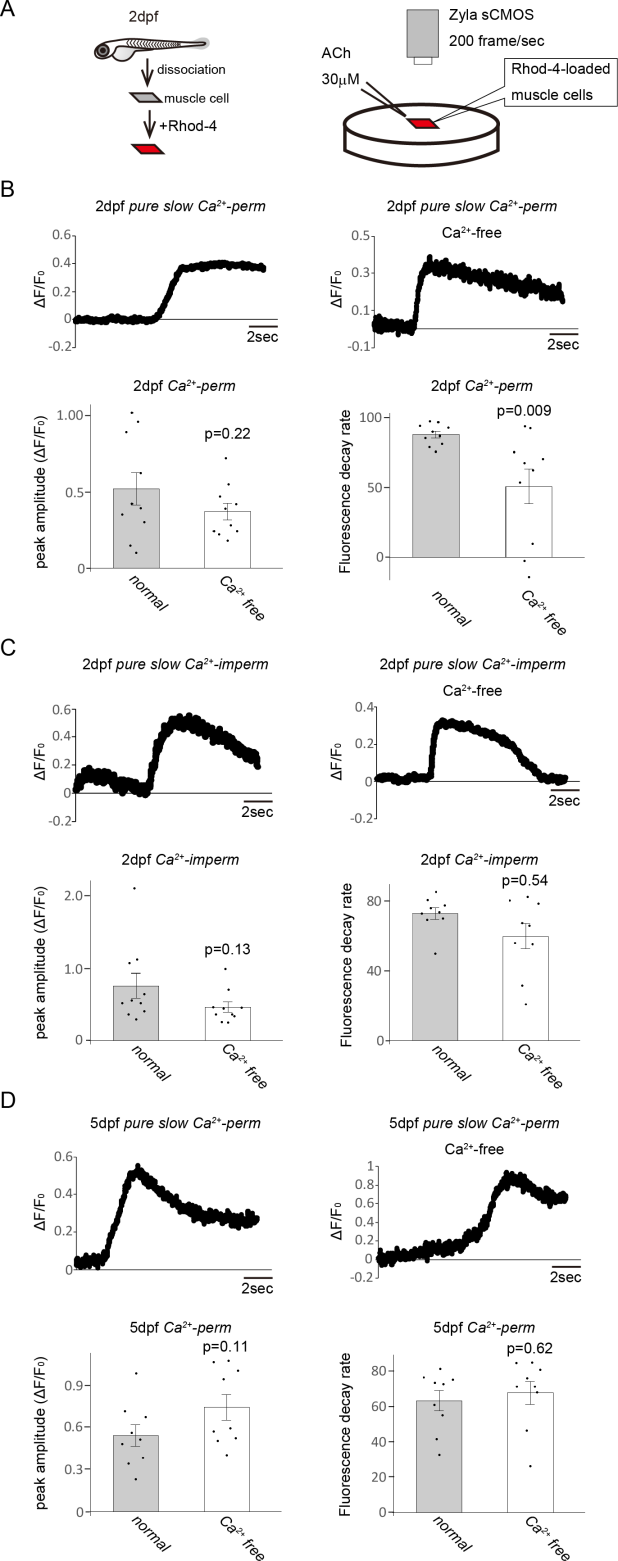
(A) Ca^2+^ response elicited by acetylcholine (ACh) in a dissociated single slow muscle cell was analysed with the fluorescent Ca^2+^ indicator Rhod-4. Ca^2+^ signals were recorded using the sCMOS camera at 200 frames s^−1^. (B) Ca^2+^ imaging for slow muscle cells of *pure slow Ca^2+^-perm* performed in normal or Ca^2+^-free Ringer’s solution. The experiments were performed in 2 dpf larvae. Representative traces showing the increase of Δ*F*/*F*_0_ in slow muscle cells in normal Ringer’s solution (left panel) and Ca^2+^-free Ringer’s solution (right panel). The peak amplitude of Δ*F*/*F*_0_ and fluorescence decay rate were calculated. At the 2 dpf stage, the fluorescence decay rate was significantly lower in muscle cells in the Ca^2+^-free solution than in muscle cells in the normal solution. There was no significant difference in the peak amplitude. (C) Ca^2+^ imaging for slow muscle cells of *pure slow Ca^2+^-imperm* performed in normal or Ca^2+^-free Ringer’s solution. The experiments were performed in 2 dpf larvae. Representative traces showing the increase of Δ*F*/*F*_0_ in slow muscle cells in normal Ringer’s solution (left panel) and Ca^2+^-free Ringer’s solution (right panel). The peak amplitude of Δ*F*/*F*_0_ and fluorescence decay rate were calculated. There was no significant difference in either peak amplitude or decay time. (D) Ca^2+^ imaging for slow muscle cells of *pure slow Ca^2+^-perm* performed in normal or Ca^2+^-free Ringer’s solution. The experiments were performed in 5 dpf larvae. Representative traces showing the increase of Δ*F*/*F*_0_ in slow muscle cells in normal Ringer’s solution (left panel) and Ca^2+^-free Ringer’s solution (right panel). The peak amplitude of Δ*F*/*F*_0_ and fluorescence decay rate were calculated. There was no significant difference in either peak amplitude or decay time.

To confirm the importance of Ca^2+^ influx through AChRs for the activities of slow muscles, the Ca^2+^ response of slow muscle cells was assessed under a Ca^2+^-free condition. We analysed the Ca^2+^ response of dissociated slow muscle cells of *pure slow Ca^2+^-perm* and *Ca^2+^-imperm* fish to ACh in Ca^2+^-free bath solution and Ca^2+^-containing (normal) bath solution with a fluorescent Ca^2+^ indicator (Rhod-4) (figure 5A). Unlike the *in vivo* Ca^2+^ imaging, in this experiment, we evaluated the kinetics of Ca^2+^ response by calculating fluorescent decay rate (Δ*F*/*F*_0_ of 3 s after the peak divided by the peak Δ*F*/*F*_0_) because Δ*F*/*F*_0_ did not reach 50% of the peak amplitude, which is arguably due to the absence of acetylcholine esterase in the bath solution. As a result, the peak amplitude of the Ca^2+^ response of cells from *pure slow Ca^2+^-perm* in Ca^2+^-free bath solution was not significantly different from that in normal bath solution at the 2 dpf stage (figure 5B; 0.52 ± 0.11 in normal bath solution, 0.37 ± 0.05 in Ca^2+^-free bath solution; *p* = 0.22). On the other hand, the fluorescence decay rate significantly decreased in the Ca^2+^-free condition (figure 5B; 87.7 ± 2.4% in *pure slow Ca^2+^-perm* in normal bath solution, 50.7 ± 12.3% in Ca^2+^-free bath solution; *p* < 0.01, *p* = 0.009). In slow muscle cells of *pure slow Ca^2+^-imperm*, both peak amplitude (figure 5C; 0.78 ± 0.18 in normal bath solution, 0.48 ± 0.08 in Ca^2+^-free bath solution; *p* = 0.13) and decay rate (figure 5C; 72.4 ± 3.3% in normal bath solution, 59.4 ± 7.2% in Ca^2+^-free bath solution; *p* = 0.54) did not change with the external Ca^2+^. In addition, the Ca^2+^ response of slow muscle cells in *pure slow Ca^2+^-perm* was unaffected by external Ca^2+^ level at the 5 dpf stage (figure 5D; peak amplitude = 0.54 ± 0.08 in normal bath solution, 0.74 ± 0.09 in Ca^2+^-free bath solution; *p* = 0.11, decay rate = 63.3 ± 5.7% in normal bath solution, 67.7 ± 6.5% in Ca^2+^-free bath solution; *p* = 0.62). Therefore, slow muscle cells require Ca^2+^ influx through AChRs for a sustained Ca^2+^ response at the early developmental stage (2 dpf). The sustained Ca^2+^ response may be important for locomotion. However, at 5 dpf, the Ca^2+^ responses of slow muscle cells in both *pure slow Ca^2+^-perm* and *Ca^2+^-imperm* shortened. Thus, slow muscle cells need a sustained Ca^2+^ response in relatively early developmental stages around 2 dpf, and the Ca^2+^ influx through AChRs plays a crucial role in prolonging the Ca^2+^ response. The Ca^2+^ influx through AChRs becomes less important by the 5 dpf stage.

### 3.5. Ca^2+^ influx through acetylcholine receptors is also important for the development of slow muscle cells

**Figure 6.**
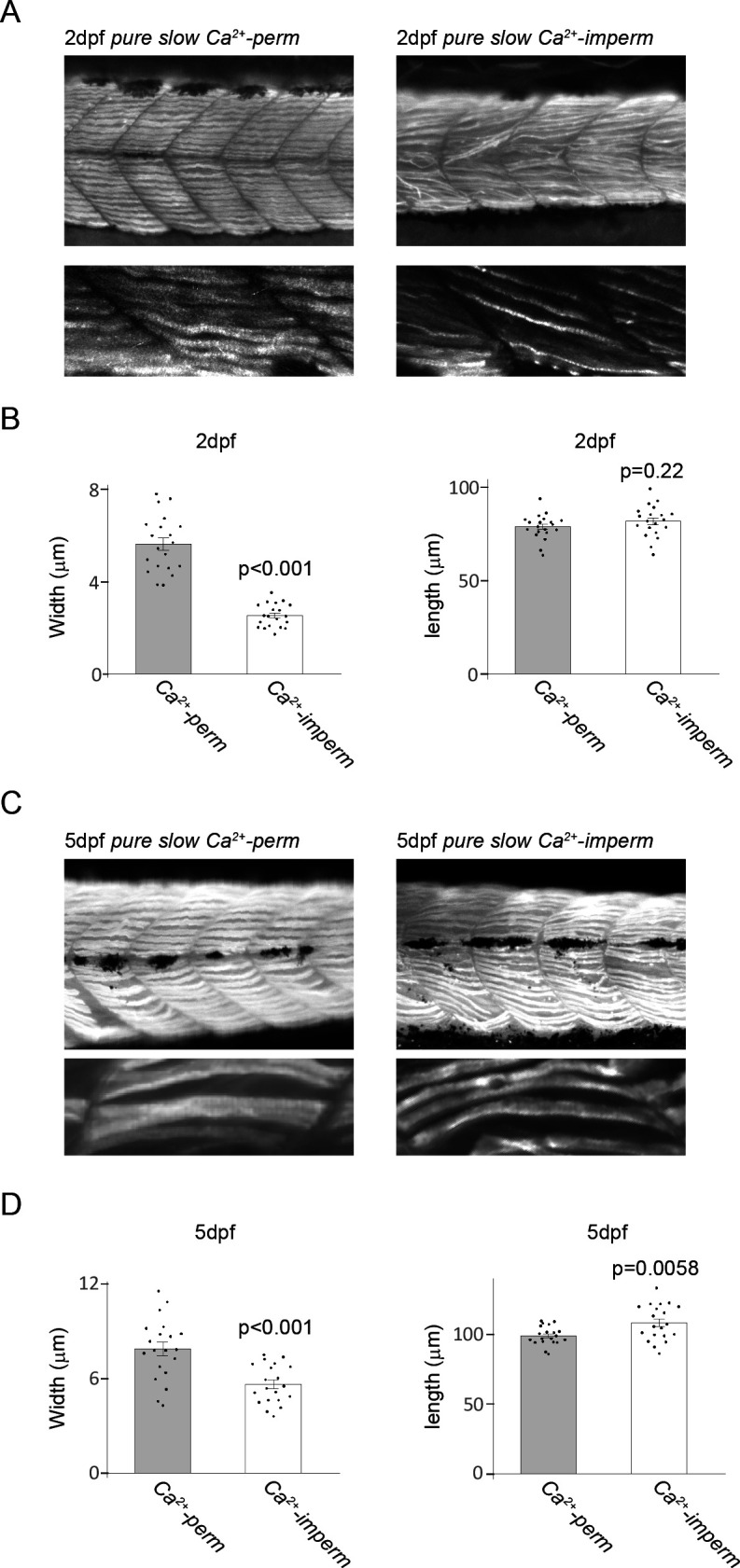
(A) Photographs showing trunk regions of a *pure slow Ca^2+^-perm* larva (2 dpf) and a *pure slow Ca^2+^-imperm* larva (2 dpf) at low magnification (upper panels) and high magnification (lower panels). Slow muscle cells were labelled with slow muscle-specific F59 antibodies. (B) The width and length of single slow muscle cells were measured. In *pure slow Ca^2+^-imperm*, slow muscle cells were significantly thinner than those in *pure slow Ca^2+^-perm*. There was no significant difference in the length of slow muscle cells between *pure slow Ca^2+^-perm* and *Ca^2+^-imperm*. (C) Photographs showing trunk regions of a *pure slow Ca^2+^-perm* larva (5 dpf) and a *pure slow Ca^2+^-imperm* larva (5 dpf) at low magnification (upper panels) and high magnification (lower panels). Slow muscle cells were labelled with slow muscle-specific F59 antibodies. (D) The width and length of single slow muscle cells were measured. In *pure slow Ca^2+^-imperm*, slow muscle cells were significantly thinner than those in *pure slow Ca^2+^-perm*. In *pure slow Ca^2+^-imperm*, slow muscle cells were significantly longer than those in *pure slow Ca^2+^-perm*.

In addition to the Ca^2+^ response, inhibition of Ca^2+^ influx through AChRs affects the morphology of the slow muscle cell. We labelled slow muscle cells of the *pure slow Ca^2+^-perm* and *Ca^2+^-imperm* lines by immunohistochemistry (figure 6). We then observed these slow muscle cells in a lateral view and measured their width and length. In 2 dpf larvae, slow muscle cells of *pure slow Ca^2+^-imperm* were significantly thinner than those of *pure slow Ca^2+^-perm* (5.62 ± 0.27 µm in *Ca^2+^-perm*, 2.52 ± 0.11 µm in *Ca^2+^-imperm*; *p* < 0.001) (figure 6B). However, there was no significant difference in their length (78.4 ± 1.5 µm in *Ca^2+^-perm*, 81.4 ± 1.9 µm in *Ca^2+^-imperm*; *p* = 0.22) (figure 6B). Although the width of *pure slow Ca^2+^-imperm* was still smaller than that of *pure slow Ca^2+^-perm* at 5 dpf (7.84 ± 0.44 µm in *Ca^2+^-perm*, 5.61 ± 0.28 µm in *Ca^2+^-imperm*; *p* < 0.001) (figure 6C,D), it had grown more than twice as thick compared to 2 dpf larvae. The length of slow muscle cells of *pure slow Ca^2+^-imperm* was larger than that of slow muscle cells of *pure slow Ca^2+^-perm* (98.5 ± 1.5 µm in *Ca^2+^-perm*, 107.8 ± 2.8 µm in *Ca^2+^-imperm*; *p* < 0.01) (figure 6D) at 5 dpf.

## 4. Discussion

### 4.1. Ca^2+^ influx through acetylcholine receptors contributes to the growth of slow muscles at early developmental stages

The results of the present study suggest that slow muscle-type AChRs of zebrafish show much higher Ca^2+^ permeability than those of fast muscle-type AChRs (figure 1). We generated a Tg line that expresses only mutated slow muscle-type AChRs with low Ca^2+^ permeability and compared its locomotor activities with those of the γεDKO line, which lacks fast muscle-type AChRs and expresses only Ca^2+^-permeable slow muscle-type AChRs (figures 2 and 3) [8]. These zebrafish lines allowed us to assess the significance of Ca^2+^ influx through AChRs in slow muscle cells with the influence of fast muscles eliminated. The results of locomotion analyses suggest that Ca^2+^ influx from AChRs plays an important role in the contraction of slow muscle cells in the early developmental stages (figure 3). Ca^2+^ imaging also showed that slow muscle cells require Ca^2+^ influx through AChRs for sufficient Ca^2+^ response to ACh at the 2 dpf stage (figures 4 and 5). In addition, morphological analysis revealed that the slow muscle cells became thinner when the Ca^2+^ permeability of their AChRs was decreased (figure 6). Inhibition of Ca^2+^ influx through AChRs delays the growth of slow muscle cells, which presumably compromises motor functions at the 2 dpf stage. The muscle force is proportional to the diameter or cross-sectional area of muscles [20]. Unexpectedly, the slow muscle fibres of *Pure slow Ca^2+^-imperm* form thick bundles by 5 dpf (figure 6C,D), which may underlie the improved locomotion.

The morphological alterations in slow muscle cells of *pure slow Ca^2+^-imperm* are consistent with the results of previous studies suggesting that intracellular Ca^2+^ signalling plays an important role in the development of slow muscle cells [21–23]. Pharmacological inhibition of AChRs or RyRs in early developmental stages induced abnormal organization in slow muscle fibres [23]. The authors suggested that the slow muscle fibres were not aligned and they could not form thick bundles. Cheung *et al*. [22] also reported that RyR antagonist treatment during early developmental stages disrupted the banding of slow muscle fibres [22]. Therefore, the elevation of intracellular Ca^2+^ levels during embryonic stages is important for the development of slow muscle cells, and the source of the Ca^2+^ is the SR. The results of the present study suggested that not only Ca^2+^ stores in the SR but also the influx of extracellular Ca^2+^ through AChRs contributes to the thickening of slow muscle cells. The Ca^2+^ influx through AChRs may be required to stimulate Ca^2+^ release from RyRs to promote the growth of slow muscle cells.

Additionally, although the mechanism has not been clarified, Brennan *et al*. [23] reported that the slow muscle fibres of mutant zebrafish lacking AChRs tend to be longer than those of wild-type zebrafish because of the abnormal organization of myofibrils [23]. They suggested that the number of sarcomeres was increased in slow muscle fibres of the mutant and hypothesized that the increase of intracellular Ca^2+^ induced by ACh stimulation might limit the sarcomere number. Interestingly, in the present study, slow muscle cells of *pure slow Ca^2+^-imperm* also became longer than those of *pure slow Ca^2+^-perm* at the 5 dpf (figure 6). Ca^2+^ influx through AChRs may also be involved in determining the sarcomere number.

Notably, both *pure slow Ca^2+^-perm* and *pure slow Ca^2+^-imperm* lines began to exhibit a thinner body shape around 7–8 dpf and died by 14 dpf. Although further investigation is needed to clarify the cause of death around 14 dpf, alterations in swim bladder formation (both lines lack a swim bladder) or atrophy of fast muscle cells lacking AChRs are possible causes of death in these lines.

### 4.2. Ca^2+^ influx through acetylcholine receptors contributes to slow muscle contraction

In the present study, locomotor activities of *pure slow Ca^2+^-imperm* were markedly decreased at the 2 dpf. One possible reason we proposed for the loss of motor function is that disruptions in the development of slow muscle cells result in thinner slow muscle fibres, which, in turn, leads to motor dysfunction. In addition to the morphological effect, we need to consider the possibility that Ca^2+^ influx through AChRs is involved in the physiological process of muscle contraction. In zebrafish, the contraction process of slow muscle cells is different from that of fast muscle cells. Slow muscle cells of zebrafish do not fire action potentials due to the lack of voltage-gated Na^+^ channels (Nav) [24]. Thus, cation influx through AChRs must activate L-type Ca^2+^ channels directly (AChRs–L-type Ca^2+^ channel pathway) to induce Ca^2+^ release from the SR. In this process, Ca^2+^ influx through AChRs not only stimulates L-type Ca^2+^ channels but may also directly activate RyRs through the Ca^2+^-induced Ca^2+^ release (CICR) mechanism, as suggested by the results of a study on the muscle cells of *Ciona* [10]*.* Ca^2+^ influx through AChRs may support Ca^2+^ release during muscle contraction by these mechanisms*.* The results of Ca^2+^ imaging in the present study showed that the inhibition of Ca^2+^ influx through AChRs results in a shortened Ca^2+^ response. Consequently, RyRs in the SR are possibly unable to release sufficient Ca^2+^ for the contractions of slow muscles without the Ca^2+^ influx through AChRs. This could be another reason for the reduced locomotor activity in *pure slow Ca^2+^-imperm* at the 2 dpf stage.

### 4.3. Ca^2+^ influx through acetylcholine receptors becomes less important for slow muscle cells at later stages

At the 5 dpf stage, slow muscle cells in both *pure slow Ca^2+^-perm* and *Ca^2+^-imperm* tended to display shorter Ca^2+^ response than that at the 2 dpf stage, and there was no significant difference in decay time between those two zebrafish lines. A possible reason for the shortened Ca^2+^ response at 5 dpf could be the development of the Ca^2+^ re-uptake system, such as SR Ca^2+^-ATPase (SERCA) and mitochondria. The expression of SERCA changes throughout the developmental process in mammals [25,26], and the expression of SERCA possibly increases between 2 and 5 dpf in zebrafish. Mitochondria also play important roles in the re-uptake of Ca^2+^ in muscle cells. In mice, mitochondria gradually shift their position within the muscle cell during development, positioning themselves close to the SR to effectively re-uptake Ca^2+^ from the SR [27]. Thus, it is possible that similar positional changes occurred in zebrafish between 2 and 5 dpf. These developments of Ca^2+^ re-uptake systems may have shortened the Ca^2+^ response in slow muscle cells at 5 dpf.

The results of the locomotor analysis suggested that the Ca^2+^ permeability of AChRs becomes less important for the contraction of slow muscles at the 5 dpf stage (figure 3). Although the slow muscle cells of *pure slow Ca^2+^-imperm* were still thinner than those of *pure slow Ca^2+^-perm*, they became thicker at 5 dpf than at 2 dpf (figure 6). Thus, the growth of slow muscle cells may have improved locomotion. The Ca^2+^ influx through AChRs may still be important for the development of slow muscle cells at 5 dpf. However, the effect of reduction in the Ca^2+^ influx on locomotor activities may be more apparent at 2 dpf, when the muscle cells are thinner.

### 4.4. Ca^2+^ signalling pathway in slow muscle cells in zebrafish development

**Figure 7.**
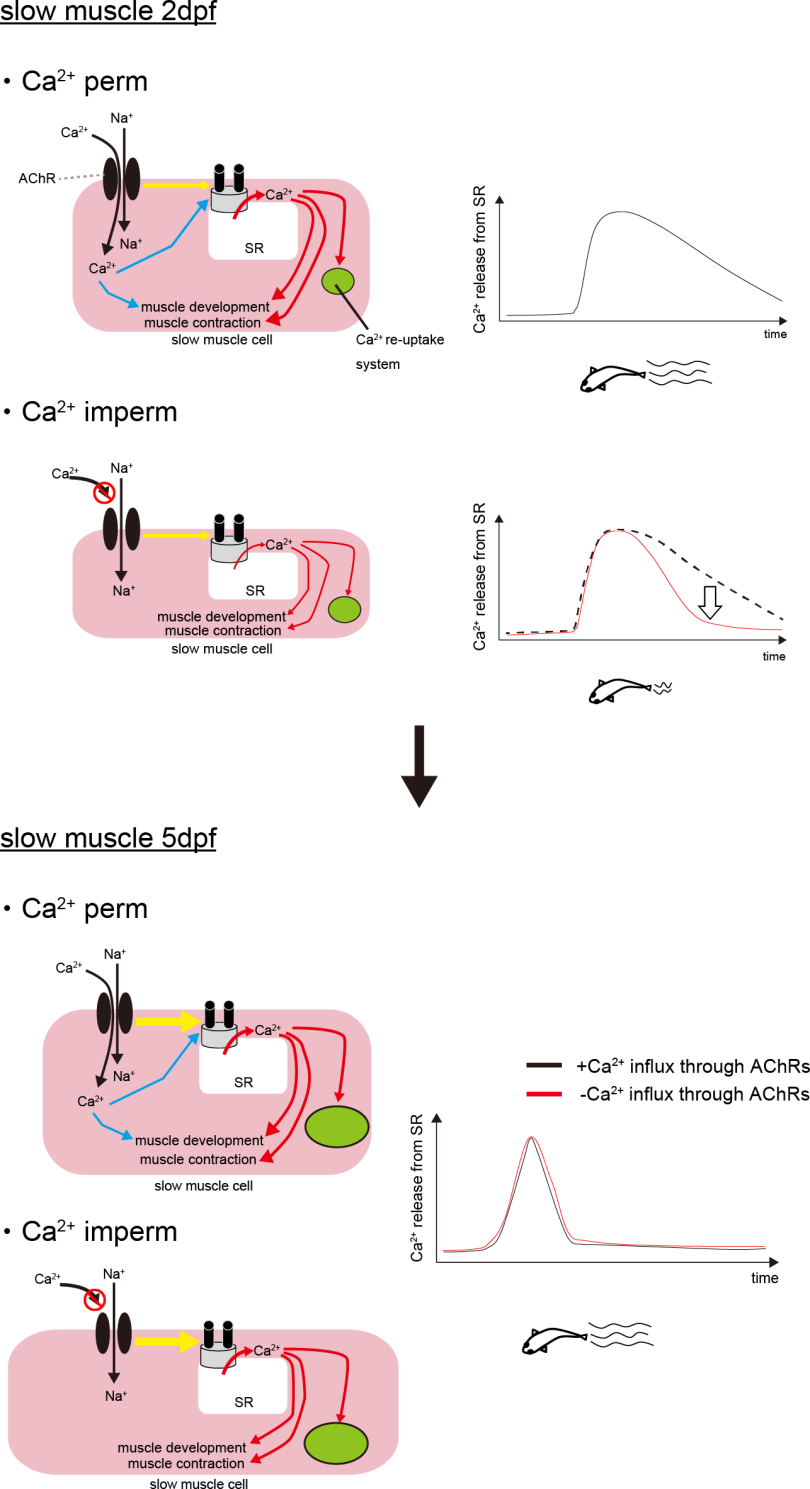
Schematic illustrations depict the functions of Ca^2+^ influx through AChRs suggested by the results of the present study. At 2 dpf, Ca^2+^ influx through AChRs stimulates the development of slow muscle cells. Thus, slow muscle cells became thinner in *pure slow Ca^2+^-imperm*. Ca^2+^ influx through AChRs also contributes to Ca^2+^ release during the contraction process by depolarizing the membrane potential or CICR system. The AChR–L-type Ca^2+^ channel pathway (yellow arrow) is still immature at the 2 dpf stage, which makes the support of Ca^2+^ influx through AChRs important for activating the L-type Ca^2+^ channel–RyR complexes efficiently. Reduction of Ca^2+^ influx through AChRs results in insufficient Ca^2+^ release, which was observed in slow muscle cells of *pure slow Ca^2+^-imperm*. At 5 dpf, the slow muscle cells are thicker than those at 2 dpf, even in *pure slow Ca^2+^-imperm*. This morphological development allows *pure slow Ca^2+^-imperm* larvae to exhibit locomotion comparable to that of *pure slow Ca^2+^-perm*. Additionally, the AChR–L-type Ca^2+^ channel pathway is well developed by this stage, decreasing the contribution of Ca^2+^ influx through AChRs to Ca^2+^ release. At the 5 dpf stage, slow muscle cells can induce sufficient Ca^2+^ release primarily through the AChR–L-type Ca^2+^ channel pathway without relying on Ca^2+^ influx through AChRs. Besides, the Ca^2+^ re-uptake system is also developed by 5 dpf, and the maturation of this re-uptake system may lead to shorter Ca^2+^ responses.

In addition to development, Ca^2+^ entry through AChRs is possibly more important for the contraction process of slow muscle cells at 2 dpf than at 5 dpf. We hypothesize that the mechanism for controlling muscle contraction is not fully developed at 2 dpf, and the immature slow muscle cells, therefore, require Ca^2+^ influx through AChRs for their functions. Based on the results of previous studies and the results of the present study, we propose that the following series of events occurs in activated immature slow muscle cells. AChRs are first stimulated by ACh from motor neurons. Na^+^ and Ca^2+^ influx through the AChRs depolarize membrane potential. L-type Ca^2+^ channels are then activated, which in turn triggers the opening of RyRs in the SR to release Ca^2+^. Note that electrophysiological analysis has shown that the L-type Ca^2+^ channel of zebrafish does not allow permeation of Ca^2+^ [28]. In this process, the Ca^2+^ influx through AChRs may contribute to both the development of slow muscle cells and membrane depolarization. Ca^2+^ entry through AChRs may also directly stimulate RyRs and thereby induce Ca^2+^ release from the SR, probably in the manner of CICR [29,30]. The released Ca^2+^ facilitates the development of slow muscle cells while inducing muscle contraction. In general, CICR is important for the contraction of cardiac muscles [31], but it is not essential for the functions of skeletal muscles. However, previous studies in amphibians suggested that CICR contributes to the contraction of skeletal muscles. In amphibians, RyRβs (corresponding to RyR3s of mammals) are localized outside the junctions between T-tubules and the SR, and the RyRβs are activated by CICR [32,33]. Besides, CICR is observed in embryonic or early postnatal myofibres of mammals. These immature myofibres express RyR3 and exhibit spontaneous Ca^2+^ sparks, which represent Ca^2+^ release from the SR, independent from functions of the L-type Ca^2+^ channel [34]. The Ca^2+^ spark is considered to be initiated by CICR because its frequency is increased by the elevation of the cytosolic Ca^2+^ level [35]. During development, Ca^2+^ sparks are rarely observed in mammalian muscle cells [36,37] because most RyRs interact with L-type Ca^2+^ channels, and spontaneous Ca^2+^ release from RyRs ceases to occur. In zebrafish, T-tubules are almost fully developed by 2 dpf [38], but the number of RyR1 contacts with the L-type Ca^2+^ channel is possibly fewer than that in matured animals at this stage. Actually, the expression of RyR1 at 1–3 dpf stages is less than that in 6 dpf larvae [39]. Therefore, the AChRs–L-type Ca^2+^ channel pathway may not be fully developed at 1–3 dpf stages. Thus, the CICR mechanism might be involved in the contractions of muscle cells with the immature AChRs–L-type Ca^2+^ channel pathway. At 5 dpf, the L-type Ca^2+^ channel–RyR complex increases, and the AChRs–L-type Ca^2+^ channel pathway becomes mature (figure 7).

## Data Availability

All data supporting the findings of this study are available within the article and its supplementary materials [40].
